# Liver-enriched transcription factors are critical for the expression of hepatocyte marker genes in mES-derived hepatocyte-lineage cells

**DOI:** 10.1186/1471-2199-10-35

**Published:** 2009-04-23

**Authors:** Pakpoom Kheolamai, Alan J Dickson

**Affiliations:** 1 Faculty of Life Sciences, The University of Manchester, Manchester, M13 9PT, UK; 2 Faculty of Medicine, Thammasat University, Pathumthani, 12120, Thailand

## Abstract

**Background:**

Induction of stem cell differentiation toward functional hepatocytes is hampered by lack of knowledge of the hepatocyte differentiation processes. The overall objective of this project is to characterize key stages in the hepatocyte differentiation process.

**Results:**

We established a mouse embryonic stem (mES) cell culture system which exhibited changes in gene expression profiles similar to those observed in the development of endodermal and hepatocyte-lineage cells previously described in the normal mouse embryo. Transgenic mES cells were established that permitted isolation of enriched hepatocyte-lineage populations. This approach has isolated mES-derived hepatocyte-lineage cells that express several markers of mature hepatocytes including albumin, glucose-6-phosphatase, tyrosine aminotransferase, cytochrome P450-3a, phosphoenolpyruvate carboxykinase and tryptophan 2,3-dioxygenase. In addition, our results show that the up-regulation of the expression levels of hepatocyte nuclear factor-3α, -4α, -6, and CCAAT-enhancer binding protein-β might be critical for passage into late-stage differentiation towards functional hepatocytes. These data present important steps for definition of regulatory phenomena that direct specific cell fate determination.

**Conclusion:**

The mES cell culture system generated in this study provides a model for studying transition between stages of the hepatocyte development and has significant potential value for studying the molecular basis of hepatocyte differentiation *in vitro*.

## Background

Liver failure is one of the major causes of morbidity and mortality worldwide [1]. The only effective treatment so far for acute and chronic liver failure is liver transplantation [2]. However, liver transplantation has several limitations, especially, the shortage of organ donors. During the last decade, hepatocyte transplantation therapy has emerged as an attractive alternative treatment for end-stage liver disease [3-5]. To enhance the potential for this approach, hepatocyte transplantation therapy requires a renewable cell source of functional hepatocytes *in vitro*. One of the most promising potential sources of functional hepatocytes is from directed embryonic stem (ES) cell differentiation.

At present, lack of detailed knowledge of the hepatocyte differentiation events restricts this potential. Several groups have reported that mouse embryonic stem (mES) cells can differentiate towards hepatocyte-lineage, based on the expression of several hepatocyte-lineage marker genes, but the underlying mechanisms that steer the induction of stem cell differentiation toward functional hepatocytes is poorly characterized [6-9]. Hepatocyte-lineage marker genes have been categorized into four groups, representing stages in the potential sequence of molecular events of hepatocyte differentiation. The first group are endodermal markers (expressed in endodermal cells, the precursor of all hepatocyte-lineage cells) including α-fetoprotein (AFP) and hepatocyte nuclear factor-3β (HNF-3β) [10]. The second group are fetal hepatocyte markers (expressed in fetal hepatocytes) including albumin [10,11]. The third group are perinatal hepatocyte markers (expressed in hepatocytes around the time of birth) including glucose-6-phosphatase (G6Pase) and tyrosine aminotransferase (TAT) [12]. The fourth group are postnatal (mature) hepatocyte markers (expressed in hepatocytes in the period following birth) including cytochrome P450-3a (Cyp3a), phosphoenolpyruvate carboxykinase (Pepck) and tryptophan 2,3-dioxygenase (TDO) [13,14].

The expression of hepatocyte-lineage marker genes is primarily regulated at transcriptional level [15,16]. Promoters of hepatocyte-lineage marker genes contain combinations of DNA regulatory elements for binding liver-enriched transcription factors (LETFs) [15,16]. Despite the importance of LETFs in regulation of expression of hepatocyte-lineage marker genes, as yet, few studies have examined the profile and sequence of expression of LETFs in the differentiation of ES cells.

The overall aim of this study was to determine the expression of LETFs during differentiation of ES cells toward hepatocyte-lineages *in vitro*. Our study had two linked stages. Firstly, we developed culture conditions that enhanced differentiation towards a hepatic phenotype. Secondly, to permit a more detailed characterization of the underlying regulatory mechanisms that define mES-derived hepatocyte-lineage cells, transgenic mES cell lines expressing green fluorescent protein (GFP), under the control of an albumin promoter/enhancer element were created and used to purify hepatocyte-lineage cells by fluorescent-activated cell sorting (FACS). The pattern of expression of hepatocyte-lineage marker and LETF genes was examined in populations of cells generated from FACS.

## Results

### HIM exhibits enhanced potential for differentiation of mES cells toward hepatocyte-lineages

**Figure 1:**
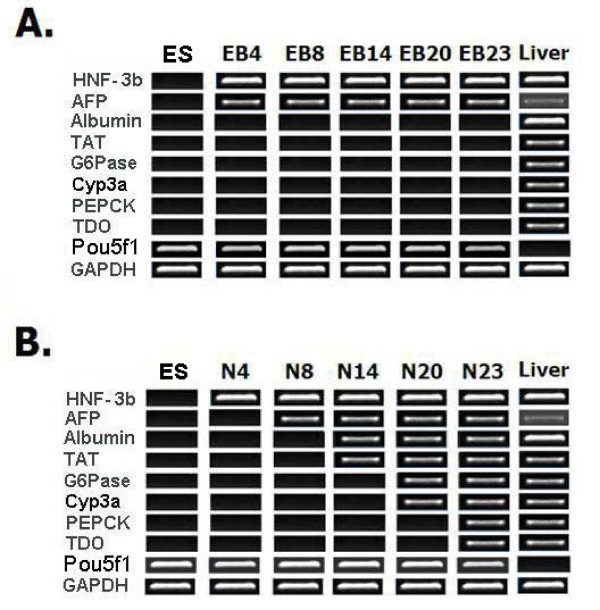
**RT-PCR analysis for hepatocyte-lineage marker genes of mES cell populations generated from NDESM and HIM cultures**. The figure shows agarose gel electrophoresis of RT-PCR analysis for HNF-3b, AFP, Albumin, TAT, G6Pase, Cyp3a, Pepck and TDO gene expression from mES cell population generated from NDESM culture (A) or HIM culture (B) over periods of culture [HNF-3b = hepatocyte nuclear factor-3β, AFP = α-fetoprotein, ALB = albumin, Cyp3a = cytochrome P450-3a, G6Pase = glucose-6-phosphatase, Pepck = phosphoenolpyruvate carboxykinase, Pou5f1 = POU domain class5 transcription factor 1, TAT = tyrosine aminotransferase, TDO = tryptophan 2,3-dioxygenase, Liver = intact mouse liver. EB = mES cell populations generated from NDESM culture, ES = non-differentiated mES cells, N = mES cell populations generated from HIM culture, Number indicate days in culture (as described in materials & methods)].

RT-PCR analysis of differentiation marker gene expression indicated that culturing of cells in hepatocyte inducing medium (HIM) supported mES cell differentiation through to postnatal stage of hepatocyte development. Populations generated from culture in non-differentiated ES medium (NDESM) without leukemia inhibitory factor (LIF) expressed endodermal marker genes (AFP and HNF-3β) but failed to show the expression of fetal (albumin), perinatal (G6Pase and TAT) or postnatal (Cyp3a and TDO) hepatocyte marker genes throughout the time course of culture (up to day 23) (Figure 1A). In contrast, mES cell populations generated from culture in HIM showed expression of all hepatocyte-lineage marker gene examined including postnatal hepatocyte markers (Figure 1B). In addition, the hepatocyte marker genes in HIM culture were expressed in an order (endodermal, fetal, perinatal, postnatal) similar to that observed in the development of the normal mouse embryo [6,17]. However, the expression of Pou5f1 was observed throughout the culture period implying that some non-differentiated mES cells were present even at the end of the culture (Figure 1).

### Flow cytometry fractionation of hepatocyte-lineage cells from differentiated mES cell populations

Although culture in HIM enhanced the derivation of cells of hepatocyte-lineage, it was clear that mES cell populations generated from this culture condition contained mixed cell types. Most mES cells (under light microscopy) exhibited circular-shaped cell morphology with a diameter about 10 μm but some (a minority) exhibited epithelial-like morphology (polygonal-shaped by microscopy, diameter about 15 μm, data not shown). The observed heterogeneity limited the interpretation of changes in gene expression in terms of differentiation toward hepatocyte-lineage. Consequently, linked to HIM culture, we isolated hepatocyte-lineage cells from the mixed population by fluorescence with EGFP expressed from a reporter construct under the control of a hepatocyte-specific (albumin) promoter/enhancer element.

**Figure 2:**
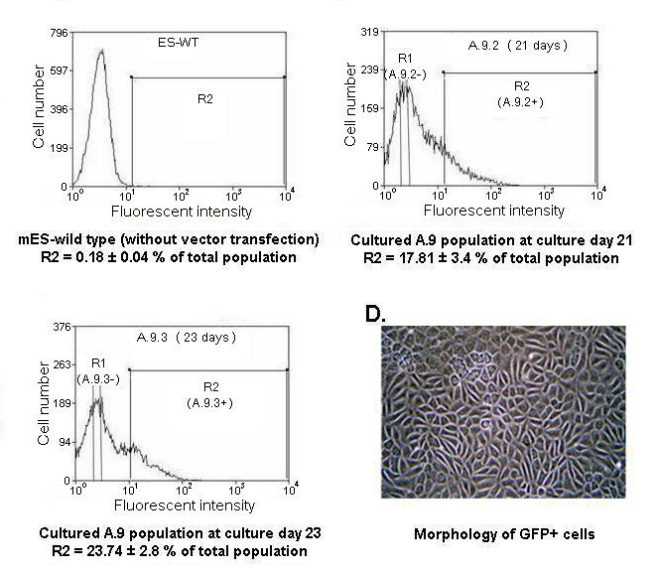
**Fluorescent-activated cell sorting (FACS) of GFP-expressing cells and GFP-negative cells from cultured A.9 populations**. The figure shows the percentage of GFP-expressing cells in cultured A.9 populations at culture day 21(B) and day 23(C). Non-transfected mES cell population (ES-WT) serve as a negative control to determine the intrinsic fluorescent intensity of the cultured mES cell populations and to define positive area R2 (A). GFP-expressing cells from R2 regions and GFP-negative cells from R1 regions of cultured A.9 populations (B-C) were sorted using a Becton Dickinson FACstar flow cytometer and CellQuest^® ^software. Data are presented as means ± SEM. (D) show the morphology of sorted GFP-expressing cells [A.9.2+ = GFP-expressing cells purified from cultured A.9 population at culture day 21, A.9.2- = GFP-negative cells purified from cultured A.9 population at culture day 21, A.9.3+ = GFP-expressing cells purified from cultured A.9 population at culture day 23, A.9.3- = GFP-negative cells purified from cultured A.9 population at culture day 23, R1 = GFP-negative cells, R2 = GFP-expressing cells].

**Figure 3:**
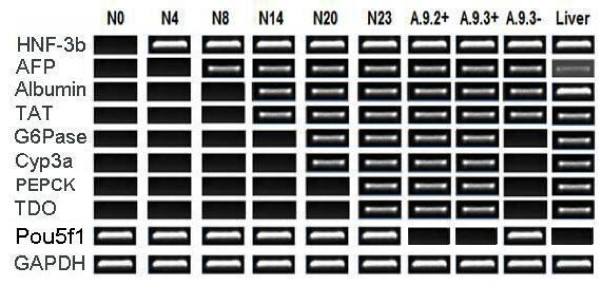
**RT-PCR analysis for hepatocyte-lineage marker genes in pALB-EGFP/ES cell line 9 (A.9) cultures**. The figure shows agarose gel electrophoresis of RT-PCR analysis for HNF-3β, AFP, Albumin, TAT, G6Pase, Cyp3a, TDO and Pepck gene expression in pALB-EGFP/ES cell line 9 (A.9) cultured in HIM [N = mES cell populations generated from HIM culture, Number indicate days in culture (as described in materials & methods), A.9.2+ = GFP-expressing cells purified from cultured A.9 population at culture day 21, A.9.3+ = GFP-expressing cells purified from cultured A.9 population at culture day 23, A.9.3- = GFP-negative cells purified from cultured A.9 population at culture day 23, HNF-3b = hepatocyte nuclear factor-3β, AFP = α-fetoprotein, Cyp3a = cytochrome P450-3a, G6Pase = glucose-6-phosphatase, Pepck = phosphoenolpyruvate carboxykinase, Pou5f1 = POU domain class5 transcription factor 1, TAT = tyrosine aminotransferase, TDO = tryptophan 2,3-dioxygenase, Liver = intact mouse liver].

After mES cell transfection, 66 clonal transfectants (pALB-EGFP/ES) were obtained and four randomly selected pALB-EGFP/ES cell lines were cultured in HIM and the percentages of GFP-expressing cells (considered here to be hepatocyte-lineage cells) were determined by flow cytometry. One cell line, A.9, expressed a higher percentage of GFP-expressing cells than the others upon differentiation and this cell line was selected for a more detail analysis of mES cell differentiation using FACS. GFP-expressing cells were purified from the A.9 populations, after 21 days (A.9.2+) and 23 days (A.9.3+) of culture by FACS and GFP-negative cells (considered here to be non-hepatocyte-lineage cells) were also isolated at each stage of culture (A.9.2- and A.9.3-, respectively) (Figure 2B–C). The percentage of GFP-expressing cells was increased from 17.81 ± 3.4% at culture day 21 to 23.74 ± 2.8% at culture day 23 (Figure 2B–C). The sorted GFP-expressing cells homogeneously exhibited epithelial-like morphology (polygonal-shaped with refractive cell border, diameter about 15 μm) (Figure 2D). Unsorted cells from cultured cell line A.9 were harvested at 23 days of culture (N23) as a further comparator. RT-PCR indicated that N23 expressed all the hepatocyte-lineage marker genes including HNF-3β, AFP, albumin, TAT, G6Pase, Cyp3a, Pepck and TDO (Figure 3). All hepatocyte-lineage marker genes detected in N23 were also detected in purified GFP-expressing cells (A.9.2+ and A.9.3+). In addition, the expression of Pou5f1 was absent in both A.9.2+ and A.9.3+ indicating that the contaminated non-differentiated mES cells were separated from the populations after FACS (Figure 3). The A.9.3- population expressed several hepatocyte-lineage marker genes (HNF-3β, AFP, albumin, TAT) but crucially did not express perinatal or postnatal marker genes (G6Pase, Pepck, Cyp3a and TDO) (Figure 3). These data indicate that the A.9 cell line cultured in HIM could generate cells with the genetic characteristics of mature hepatocytes, which by sorting allow for subsequent definition of the relationships between cell line development and transcriptional regulatory events.

**Figure 4:**
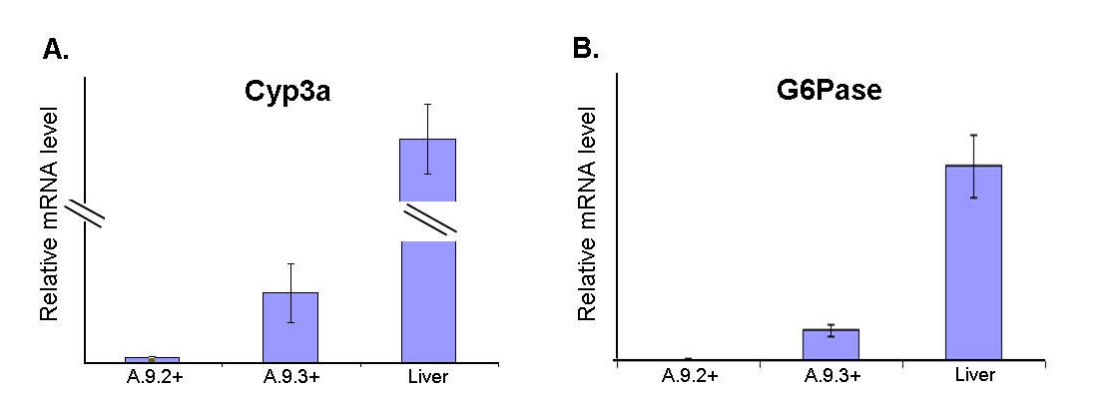
**The expression of Cyp3a and G6Pase genes in A.9.2+ and A.9.3+ populations**. The figure shows the expression level of Cyp3a (A) and G6Pase (B) genes in A.9.2+, A.9.3+ populations. Data are presented as means ± SEM of three independent experiments. (A.9.2+ = GFP-expressing cells purified from cultured A.9 population at culture day 21, A.9.3+ = GFP-expressing cells purified from cultured A.9 population at culture day 23, Cyp3a = cytochrome P450-3a, G6Pase = glucose-6-phosphatase, Liver = intact mouse liver). [*P < 0.05 vs. A.9.2+].

As a further step towards understanding regulatory events linked to mature hepatocyte differentiation processes, the expression of cytochrome P450-3a (Cyp3a) and glucose-6-phosphatase (G6Pase) was measured in A.9.2+ and A9.3+ cells by quantitative real-time PCR (qRT-PCR). Expression of Cyp3a and G6Pase increased from 21 to 23 days of culture in the GFP-expressing populations towards the level found in intact mouse liver (about 2.4 and 15.4% of the intact liver value, respectively) (Figure 4).

### Expression of liver-enriched transcription factors during mES cell differentiation

**Figure 5:**
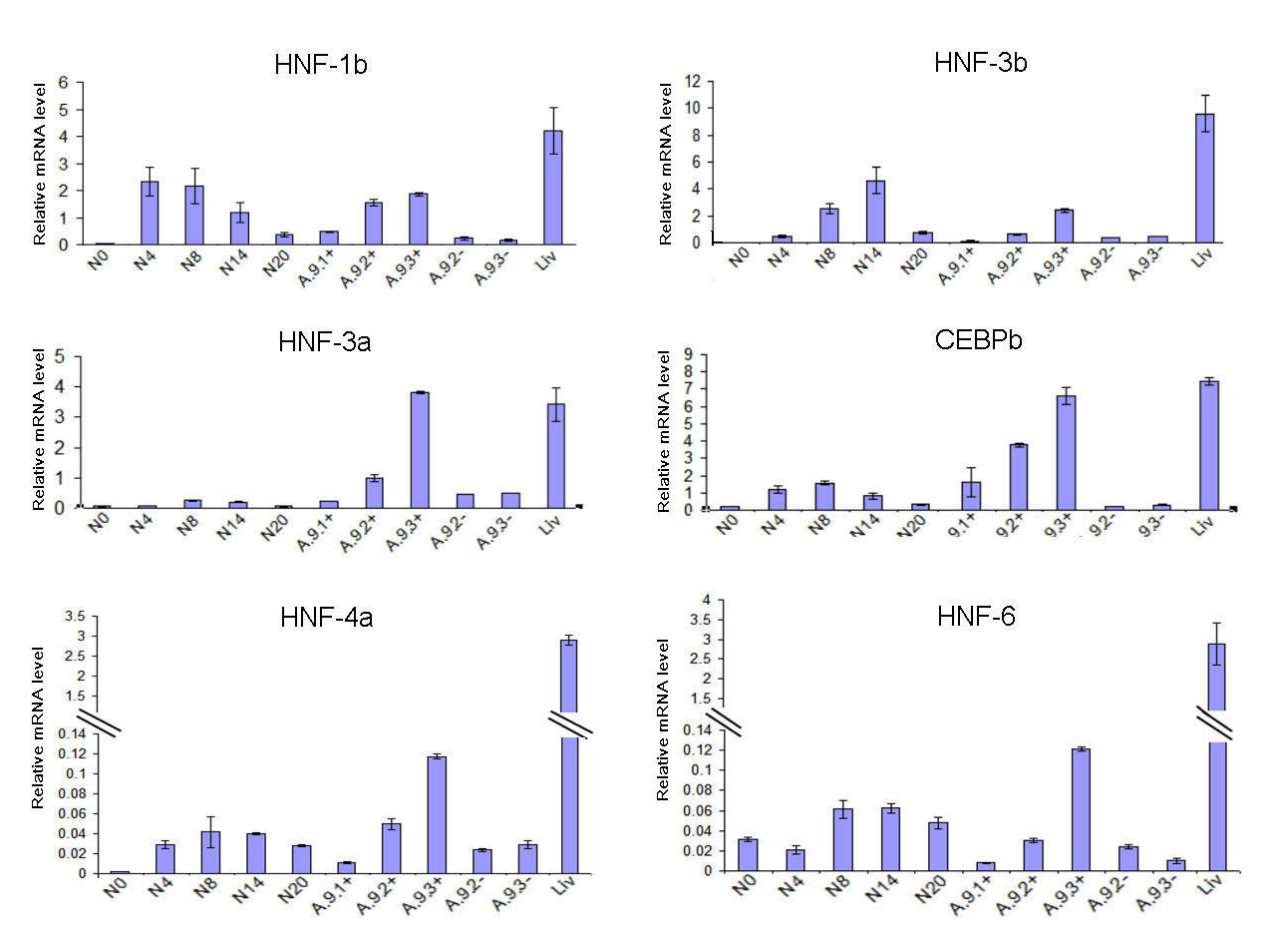
**The expression of liver-enriched transcription factor genes in pALB-EGFP/ES cell line 9 (A.9) cultures**. The figure shows the expression levels of HNF-1b (A); HNF-3b (B); HNF-3a (C); C/EBP-b (D); HNF-4a (E) and HNF-6 (F) genes in pALB-EGFP/ES cell line 9 (A.9) cultured in HIM. Data are presented as means ± SEM of three independent experiments (A.9.2+ = GFP-expressing cells purified from cultured A.9 population at culture day 21, A.9.3+ = GFP-expressing cells purified from cultured A.9 population at culture day 23, A.9.3- = GFP-negative cells purified from cultured A.9 population at culture day 23, HNF-1b, HNF-3b, HNF-3a, HNF-4a, HNF-6 = hepatocyte nuclear factor-1β, -3β, -3α, -4α and 6, respectively; C/EBP-b = CCAAT-enhancer binding protein-β, Liver = intact mouse liver). [*P < 0.05 vs. N populations].

Having defined a method for enhancement of differentiation towards hepatocyte-lineage (culture in HIM) and a means to enrich hepatocyte-lineage cells, we sought to examine how control of differentiation towards hepatocyte lineage was related to the pattern of expression of the network of LETFs. To this end we determined expression of 7 LETFs at mRNA level throughout mES culture in HIM and in populations separated on the basis of pALB-EGFP expression (Figure 5)

Expression of HNF-1β in mES cell populations was greatest (about 69% of intact mouse liver value) at day 4 of culture and remained constant over a further 10 days before decreasing (to about 8% of intact mouse liver value) (Figure 5A). The expression of HNF-3β increased over the first 10 days of culture and reached its highest value (about 48% of intact mouse liver value) at day 14 before decreasing (to about 8% of intact mouse liver value) (Figure 5B). HNF-3α and C/EBP-β exhibited a pattern of expression that was similar to that of HNF-3β, increasing over the first 8 days of culture to reach their highest values (about 8% and 21% of intact mouse liver value, respectively) at day 8 before decreasing (to about 2% and 4% of intact mouse liver value, respectively) (Figures 5C–D).

Although the expression of HNF-4α and HNF-6 also increased over the first 8 days of culture, to reach its greatest expression at that time (about 1% and 2% of intact mouse liver value, respectively) the expression of these two transcription factors remained unchanged from that point until the end of culture (Figures 5E–F). In contrast to all other LETF genes examined, C/EBP-α mRNA could not be detected at any stage of culture (despite ready detection in samples from intact mouse liver, data not shown).

GFP-expressing cell populations were isolated from A.9 cells cultured in HIM for 21 (A.9.2+) and 23 (A.9.3+) days. It was notable that the expression of all LETFs examined increased progressively from 21 to 23 days of culture for GFP-expressing populations (Figures 5A–F). In particular, the expression of HNF-3α and C/EBP-β in the A.9.3+ population reached values similar to those observed for intact mouse liver (Figures 5C–D). In contrast to HNF-3α and C/EBP-β, the expression of HNF-4α and HNF-6 in the A.9.3+ population was still much lower than that of intact mouse liver (about 4% of intact mouse liver value, in each case) (Figures 5E–F). Again, C/EBP-α mRNA expression was undetectable (data not shown).

For GFP-negative cell populations isolated after 21 and 23 days of culture (A.9.2- and A.9.3-), the expression of HNF-1β, HNF-3β, HNF-6 and C/EBP-β was similar to that of non-differentiated mES cells (N0) (Figures 5A, B, D, F) whereas the expression of HNF-3α and HNF-4α were similar to that of the unsorted albumin-negative cultured mES cell population (N8) (Figure 5C, E).

## Discussion

**Figure 6:**
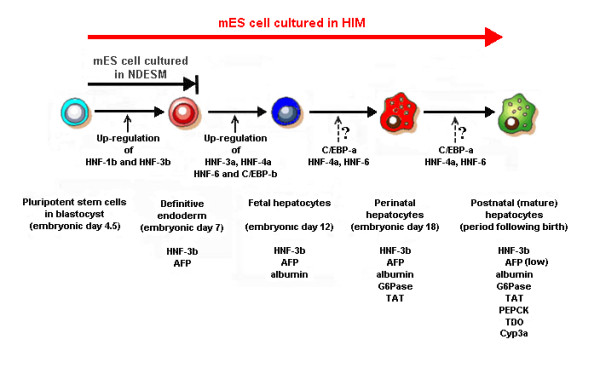
**Progression of cultured mES cells towards hepatocyte-lineages**. The figure shows the progression of mES cells in NDESM and HIM cultures along the hepatocyte-lineages. [AFP = α-fetoprotein, HNF-1b, HNF-3b, HNF-3a, HNF-4a, HNF-6 = hepatocyte nuclear factor-1β, -3β, -3α, -4α and 6, respectively; C/EBP-a, C/EBP-b = CCAAT-enhancer binding protein-α, -β, respectively; TAT = tyrosine aminotransferase, Cyp3a = cytochrome P450-3a, Pepck = phosphoenolpyruvate carboxykinase, TDO = tryptophan 2,3-dioxygenase, (low) = the expression level is low].

The ability of ES cells to differentiate to various cells of ectoderm, mesoderm and endoderm lineages *in vitro *offers a promising system to study cell differentiation processes that opens the potential for cell replacement therapy. In this study, we categorized the hepatocyte-lineage marker genes into four groups (endodermal, fetal hepatocyte, perinatal hepatocyte and postnatal hepatocyte) according to the expression pattern during the development of mouse liver [10-14,18,19] to facilitate the identification of the corresponding developmental state of the differentiated mES cells. We demonstrated that even though the differentiated EBs can generate endodermal cells (precursor of all hepatocyte-lineages), these mES-derived endodermal cells cannot proceed past a specific "check point" to generate hepatocyte-like cells (which express fetal, perinatal and postnatal hepatocyte marker genes) without the supplementation of some undetermined critical factors (Figure 6).

Several previously published papers have reported that mES cell-derived EBs expressed ALB and TAT genes, when cultured in same medium as our control condition, NDESM [9,20,21]. There are several potential reasons that would explain the apparent discrepancy. Firstly, the mES cell lines used in this study is different from those used in previous publications. It is well known that different mES cell lines have varied differentiation capacity even though cultured in the same conditions [22-24]. Secondly, unlike previous studies, we did not use the "hanging drop method" for generating EBs and this is an important factor which might contribute to the differences in the experimental results.

This is the first report, to our knowledge, to demonstrate that a relatively simple medium, supplemented with insulin and dexamethasone and no other growth factors, can support induction of cultured mES cells towards endodermal and hepatocyte-lineage cells in a pattern similar to that of mouse embryos [6,17] (Figure 6). Other workers have reported a need to make sequential additions of several growth factors to mimic the expression profile of hepatocyte-lineage markers in cultured mES cells populations but in our system this was unnecessary [6,21,25-27]. However, the percentage of albumin-expressing cells generated from HIM culture was still lower than those reported using the sequential addition of growth factors. This implies that the sequential addition of growth factors may improve the differentiation efficiency even through they are not critical for the induction of the expression of those hepatocyte-lineage genes.

It also appeared that albumin-expressing cells were also detected in GFP-negative cell populations (A.9.2- and A.9.3-) as determined by RT-PCR. This might arise through two reasons. Firstly, the albumin promoter/enhancer region used in this study (Alb-p/e) contained only parts of the whole physiological albumin promoter. Thus, it is possible that the GFP-negative, albumin-expressing cells may produce albumin-mRNA under control of the albumin promoter regions which are located outside Alb-p/e. Secondly, it is also possible that the transfected Alb-p/e might be inactivated in some albumin-expressing A.9 cells. Such events can account for the lack of GFP-expression in albumin-expressing cells.

We have shown that up-regulation of the expression of HNF-3α, HNF-4α, HNF-6, and C/EBP-β was specific to the hepatocyte-lineage cells in the heterogeneous cultured mES cell populations (Figure 5C–F). By implication, this suggests that these transcription factors might be required for the initiation of cell progression towards full expression of hepatocyte-lineage marker genes in mES-derived hepatocyte-like cells. In contrast, the expression of HNF-1β and HNF-3β in the mES-derived hepatocyte-lineage cells (which expressed fetal, perinatal and postnatal hepatocyte markers) was not significantly different from that of albumin-negative cell populations (N4, N8, which expressed only endodermal marker genes). These data suggest that although HNF-1β and HNF-3β are required for expression of endodermal marker genes (AFP) they might not be required for the expression of fetal, perinatal and postnatal hepatocyte marker genes (albumin, TAT, G6Pase, Cyp3a, Pepck and TDO) (Figure 6).

Others have reported that expression of HNF-1β and HNF-3β was increased when definitive endoderm (the precursor of all hepatocyte lineage cells) was generated and then decreased (in case of HNF-1β) or remained constant (in case of HNF-3β) during the remainder of liver development [28].

Although expression of HNF-3α and C/EBP-β in the A.9.3+ population (which is the highest of all cultured mES cell populations generated in this study) was similar to that of intact mouse liver, the expression of HNF-4α and HNF-6 was 40-fold less than that of intact liver. The expression of C/EBP-α, which is considered to be involved in perinatal and postnatal stages of hepatocyte differentiation processes [16,29-31], was not detected in any cultured mES cells populations in this study. Moreover, although A.9.2+ and A.9.3+ mES-derived cells expressed all hepatocyte-lineage marker genes examined, the expression of perinatal and postnatal hepatocyte marker genes (G6Pase and Cyp3a) was much less than that of intact mouse liver (Figure 4). The lack of the significant expression of "late phase" transcription factors such as C/EBP-α (and HNF-4α and HNF-6) implies a regulatory point of key significance towards the transition to expression perinatal and postnatal hepatocyte marker genes (G6Pase and Cyp3a) in the mES-derived hepatocyte-lineage cell populations. This interpretation agrees with reports that *C/EBP-α *-/- mice died within 8 hours after birth due to hypoglycemia caused by low expression of several glucose metabolism enzymes including G6Pase (perinatal hepatocyte marker gene) and Pepck (postnatal hepatocyte marker gene) [30,31].

## Conclusion

We have provided a detailed analysis of the sequential and coordinated changes in key transcription factor gene expression in an *in vitro *model of hepatocyte differentiation (Figure 6). These data indicate that the regulation of expression of late phase transcription factors is critical for optimization of differentiation of ES cells towards a full hepatocyte phenotype *in vitro*. However, the direct evidence for any causal relationship between specific transcription factors and the differentiation would only arise through further experiments involving over-expression and/or knockdown of specific transcription factors. Nonetheless, the system we have developed and the molecular characterization of the transcription factor (and target gene) profiles offer a critical model for direct examination of molecular determinants of hepatocyte differentiation control. That information will not only develop further understanding of cell differentiation processes but will aid researchers in examination of the potential for use of human ES cells in the generation of donor hepatocytes for cell replacement therapies.

## Methods

### Culture of mES cells

Mouse embryonic stem (mES) cells [E14, a generous gift from Dr. Christopher Ward, The University of Manchester [23]] were maintained as described previously [6]. Briefly, non-differentiated mES cells were cultured on a gelatin-coated flask [prepared by coating a tissue culture flask with 0.1% (w/v) Type A gelatin from porcine skin (Sigma, UK)] in LIF-NDESM which is Knockout™ Dulbecco's modified Eagle's medium (Gibco, UK) supplemented with 10% (v/v) fetal bovine serum (Cambrex, UK), 1% (v/v) 10 mM non-essential amino acids solution (Cambrex, UK), 1% (v/v) 200 mM L-glutamine (Cambrex, UK), 0.001% (v/v) 14.3 M β-mercaptoethanol (Sigma, UK) and 1000 unit/ml leukemia inhibitory factor (LIF, Chemicon, UK). To induce differentiation, mES cells were removed from gelatin-coated flasks by incubation with 0.25 mg/ml trypsin and 0.2 mg/ml EDTA (Cambrex, UK) for 2 minutes, trypsinized to a single cell suspension and transferred to bacterial Petri dishes (Falcon, UK) at a concentration of 2 × 10^6 ^cells/10 cm dish in NDESM without LIF and incubated at 37°C in 5% CO_2 _incubator for 4 days. During this time embryoid bodies (EBs) formed. At this stage (which was counted as culture day 0), EBs were plated and attached to gelatin-coated flasks in non-differentiating ES medium (NDESM) or hepatocyte-inducing medium (HIM) [NDESM without LIF supplemented with 1 × 10^-6 ^M dexamethasone (Sigma, UK), 1 × 10^-8 ^M insulin (Sigma, UK) and 200 mM L-glutamine (Cambrex, UK)] and allowed to attach at 37°C in 5% CO_2 _incubator for 2 days. The appropriate culture medium was replaced every 24 hours.

### pALB-EGFP Plasmid Construction

A plasmid was constructed with a 2,335 bp fragment of the mouse albumin promoter/enhancer (incorporating a region from -8.5 to -10.4 kb of the albumin enhancer fused to the -0.3 kb minimal albumin promoter sequences, a generous gift from Professor Richard Palmiter, University of Washington [32], cloned into a promoterless EGFP vector, pd2EGFP-1 (BD Biosciences Clontech, UK). The resulting construct was named pALB-EGFP. Establishment of pALB-EGFP stable transfectants of mES cells (pALB-EGFP/ES) was performed as described previously [27]. Briefly, 1 × 10^7 ^non-differentiated mES cells (maintained in LIF-NDESM) were transfected with pALB-EGFP plasmid using electroporation. After electroporation and selection, pALB-EGFP-transfected mES clones were harvested and screened using genomic PCR with EGFP-specific primers. The resultant pALB-EGFP/ES cell lines were maintained in the presence of 350 μg/ml G418 (Gibco, UK).

### Fluorescence-Activated Cell Sorting (FACS)

pALB-EGFP/ES cells were removed from culture flasks by incubation with 0.25 mg/ml trypsin and 0.2 mg/ml EDTA (Cambrex, UK) for 2 minutes and trypsinized to single cell suspensions. Suspensions (containing 0.5–1 × 10^6 ^cells) were centrifuged at 13,000 g for 1 minute and pellets were resuspended in 500 μl FACS buffer (PBS containing 2% (v/v) fetal bovine serum and 0.002% (w/v) sodium azide). Populations were analyzed and sorted using a Becton Dickinson FACstar flow cytometer and CellQuest^® ^software (BD Biosciences, UK). Non-transfected mES cells (ES-WT) were negative controls of the intrinsic fluorescent intensity of the cultured mES cell populations and defined the positive area for cell sorting.

### Reverse transcriptase-polymerase chain reaction

RNA was extracted from cells using TRIZOL^® ^(Invitrogen, UK) or RNeasy^® ^Mini Kit (Qiagen, UK) and was treated with RNase-free DNase (Sigma, UK). cDNA was synthesized from 1 μg of RNA using an oligo d(T)12–18 primer (Invitrogen, UK) and M-MLV reverse transcriptase (Roche, UK). PCR was performed using Taq DNA polymerase (Roche, UK): 94°C for 5 minutes followed by 40 cycles of denaturation (94°C, 1 minute), annealing (Table 1, 1 minute), extension (72°C, 2 minutes) and a final extension at 72°C for 10 minutes. Products were separated by electrophoresis on 1% (w/v) agarose gels. All primers used in this study (Table 1) were designed using Primer-3 output program (Whitehead Institute for Biomedical Research, 1998) and were checked by performing Basic Local Alignment Search Tool (BLAST) within the nucleotide databases for mouse genome to ensure their specificity.

**Table 1:** Target genes, primer sequences, product sizes and primer-specific annealing temperatures (AT) used for PCR

Target genes	Sequences of primer (5'-3')	Product sizes (kB)	AT (°C)
AFP (NM_007423.3)			
*Forward*	cactgctgcaactcttcgta	300	55
*Reverse*	ctttggaccctcttctgtga		
			
Albumin (NM_009654.1)			
*Forward*	tgaactggctgactgctgtg	718	55
*Reverse*	catccttggcctcagcatag		
			
HNF-3β (NM_010446.1)			
*Forward*	actggagcagctactacg	169	55
*Reverse*	cccacataggatgacatg		
			
Cyp3a (NM_007818.2)			
*Forward*	tacagcatggatgtgatca	380	55
*Reverse*	tcatacccagcaaaaataaa		
			
G6Pase (NM_008061.2)			
*Forward*	caggactggttcatcctt	210	55
*Reverse*	gttgctgtagtagtcggt		
			
Pepck (NM_011044.1)			
*Forward*	tctgccaaggtcatccagg	290	60
*Reverse*	gttttggggatgggcactg		
			
TAT (NM_146214.1)			
*Forward*	accttcaatcccatccga	206	57
*Reverse*	tcccgactggataggta		
			
TDO (NM_019911.2)			
*Forward*	agagccagcaaaggaggac	500	55
*Reverse*	ctgtctgctcctgctctgat		
			
Pou5f1 (NM_013633.2)			
*Forward*	ggcgttctctttggaaaggtgttc	313	55
*Reverse*	ctcgaaccacatccttctct		

Abbreviation: HNF-3b = hepatocyte nuclear factor-3β, AFP = α-fetoprotein, Cyp3a = cytochrome P450-3a, G6Pase = glucose-6-phosphatase, Pepck = phosphoenolpyruvate carboxykinase, Pou5f1 = POU domain class5 transcription factor 1, TAT = tyrosine aminotransferase, TDO = tryptophan 2,3-dioxygenase, GAPDH = glyceraldehyde-3-phosphate dehydrogenase.

### Quantitative real-time PCR (qRT-PCR)

MJ-white 96 well plates (Bio-Rad, UK) were used for qRT-PCR reactions. Each well contained 1–2 μl of cDNA, 5 μl 1.2 μM appropriate forward and reverse primer mix and 10 μl DyNAmo™ SYBR^® ^Green reaction mix (FINNZYME, Finland). The plate was sealed with Microseal 'B' clear adhesive film (Bio-Rad, UK) to prevent the evaporation of reactant. PCR was performed using an Opticon Monitor qRT-PCR thermal cycler (Bio-Rad, UK): 95°C initial denaturation for 10 minutes followed by 40 cycles of denaturation (94°C, 10 seconds), annealing (60°C, 20 seconds), extension (72°C, 20 seconds) followed by denaturation (76°C, 1 second). The quantity of a target gene was calculated by normalization with glyceraldehyde-3-phosphate dehydrogenase (GAPDH), using the following formula: normalized target gene quantity = 2^{-[C(T) target gene/C(T) GAPDH gene]}. The 'C(T)' value was the number of reaction cycles required for the target gene to enter logarithmic amplification. For quantification, a standard curve for each gene was calculated from 3 dilutions of the cDNA template by plotting the log value of the starting concentration versus the threshold value. Primers are specified in Table 2.

**Table 2:** Target genes, product sizes and primer sequences used for quantitative real-time PCR

Target genes	Sequences of primer (5'-3')	Product sizes (kB)
CEBP-α (NM_007678.2)		
*Forward*	gctttttgcacctccaccta	170
*Reverse*	ccacaaagcccagaaaccta	
		
CEBP-β (NM_009883.1)		
*Forward*	caagctgagcgacgagtaca	157
*Reverse*	cagctgctccaccttcttct	
		
HNF-1β (NM_009330.2)		
*Forward*	gacactcctcccatcctcaa	156
*Reverse*	ctccctctgggggatattgt	
		
HNF-3α (NM_008259.2)		
*Forward*	cagcacaagctggacttcaa	173
*Reverse*	agcacgggtctggaatacac	
		
HNF-3β (NM_010446.1)		
*Forward*	taagcgagctaaagggagca	176
*Reverse*	agagaaggggtggttgaagg	
		
HNF-4α (NM_008261.2)		
*Forward*	agaggttctgtcccagcaga	169
*Reverse*	atccagaaggagttcgcaga	
		
HNF-6 (NM_008262.2)		
*Forward*	ctgtgaaactcccccaggta	179
*Reverse*	tgaaactaccgctcacgttg	
		
GAPDH (NM_199472.1)		
*Forward*	acccagaagactgtggatgg	172
*Reverse*	cacattgggggtaggaacac	

Abbreviation: HNF = hepatocyte nuclear factor, C/EBP = CCAAT-enhancer binding protein, GAPDH = glyceraldehyde-3-phosphate dehydrogenase

### Statistical analysis

Data are presented as means ± standard error of the mean (SEM) for the number of individual cell preparations identified. In some cases one measurement in each experiment was set at 100% as indicated in the text. All other values were calculated relative to this. The paired Student's t-test was used to assess the significance of differences between observed data. P < 0.05 was considered to be statistically significant.

## Abbreviations

**mES**: Mouse embryonic stem cell; **AFP**: α-fetoprotein; **HNF**: hepatocyte nuclear factor; **C/EBP**: CCAAT-enhancer binding protein; **G6Pase**: Glucose-6-phosphatase; **TAT**: tyrosine aminotransferase; **Cyp3a**: cytochrome P450-3a; **Pepck**: phosphoenolpyruvate carboxykinase; **Pou5f1**: POU domain class5 transcription factor 1; **TDO**: tryptophan 2,3-dioxygenase; **LETFs**: liver-enriched transcription factors; **GFP**: green fluorescent protein; **FACS**: fluorescent-activated cell sorting; **HIM**: hepatocyte inducing medium; **NDESM**: non-differentiated ES medium; **LIF**: leukemia inhibitory factor; **EBs**: embryoid bodies; **pALB-EGFP/ES**: pALB-EGFP-transfected mES cell line; **ES-WT**: Non-transfected mES cells; **qRT-PCR**: Quantitative real time PCR; **GAPDH**: Glyceraldehyde-3-phosphate dehydrogenase; **SEM**: standard error of the mean.

## Authors' contributions

PK carried out the experiments, performed data analysis and participated in the sequence alignment and drafted the manuscript. AJD designed and supervised the study and helped to draft the manuscript. All authors read and approved the final manuscript.
